# On-Treatment Elevation in Hepatic Transaminases during HCV Treatment with Ombitasvir, Paritaprevir, Dasabuvir, Ritonavir, and Ribavirin: A Case Series

**DOI:** 10.1155/2016/6151570

**Published:** 2016-05-26

**Authors:** Madelyne Bean, Lydia Tang, Shyam Kottilil, Kimberly L. Beavers, Eric G. Meissner

**Affiliations:** ^1^Division of Infectious Diseases, Department of Medicine, Medical University of South Carolina, 135 Rutledge Avenue Suite 1209, MSC 752, Charleston, SC 29425, USA; ^2^Department of Pharmacy Services, Medical University of South Carolina, 150 Ashley Avenue, Charleston, SC 29425, USA; ^3^Institute of Human Virology, University of Maryland School of Medicine, 725 West Lombard Street, Baltimore, MD 21201, USA; ^4^Division of Gastroenterology and Hepatology, Department of Medicine, Medical University of South Carolina, 114 Doughty Street Suite 249, MSC 702, Charleston, SC 29425, USA

## Abstract

Eradication of chronic hepatitis C virus (HCV) infection is now possible with all oral antiviral medications, including the combination of ombitasvir, paritaprevir, dasabuvir, and ritonavir (PrOD) with or without ribavirin. While high rates of sustained virologic response (SVR) can be achieved, a small subset of patients experience on-treatment liver enzyme elevations, in particular women using concurrent estradiol-containing oral contraceptive medications (OCPs). Herein, we describe four cases of liver enzyme elevations within 2-3 weeks of PrOD initiation in African-American men infected with HCV genotype 1a or 1b. Three patients with varying degrees of hepatic fibrosis received a full treatment course without medication modification, achieved SVR, and experienced resolution of liver enzyme abnormalities. One patient with cirrhosis was switched mid-treatment to an alternate HCV regimen, experienced subsequent resolution of liver enzyme abnormalities, and achieved SVR. In summary, these cases suggest that all HCV patients treated with PrOD, independent of gender or concurrent medications, should have laboratory monitoring for liver enzyme elevations, with a particular emphasis on early monitoring in cirrhotic patients.

## 1. Introduction

Eradication of chronic hepatitis C virus (HCV) infection is now possible with all oral regimens composed of directly acting antiviral (DAA) agents. The combination of ombitasvir (an HCV NS5A inhibitor), paritaprevir (an NS3/4A protease inhibitor), dasabuvir (a nonnucleoside NS5B polymerase inhibitor), and ritonavir (collectively referred to as PrOD) with or without ribavirin is FDA approved for treatment of genotype 1a/1b in patients with HCV monoinfection and HIV/HCV coinfection [1]. High rates of sustained virologic response (SVR) are routinely achieved, with treatment duration and concomitant ribavirin use dependent upon genotype-1 subtype and presence of cirrhosis [2–5].

An increase in serum levels of aspartate transaminase (AST) and alanine transaminase (ALT) greater than 5 times the upper limit of normal (ULN) has been observed in a minority (~1%) of patients treated with PrOD in clinical trials [1]. Women taking concomitant estradiol-containing oral contraceptive pills (OCPs) during PrOD treatment have thus far been the primary identified risk group for liver enzyme elevation [1]. In HCV monoinfected subjects, on-treatment AST/ALT elevations were noted to be typically asymptomatic, with onset in the first 4 weeks of treatment and decline within 8 weeks during continued treatment [1]. In a small study of 63 HIV/HCV patients, only 1 patient treated with PrOD and ribavirin for 24 weeks had an AST value greater than 5 times the ULN, while 15 of 17 patients with bilirubin increases 3–10x ULN were taking concomitant atazanavir for HIV [5].

Although hepatic transaminase elevation during PrOD treatment is well reported, there are as yet no published reports describing in detail the temporal changes in AST/ALT that can occur to help guide expectations for clinicians and aid in patient management. With the recent report of hepatic decompensation in a subset of cirrhotic patients treated with PrOD, particularly those with more advanced cirrhosis [6], understanding the clinical characteristics of this uncommon side effect is imperative. Here, we describe the detailed clinical course for 4 African-American male patients treated with PrOD with or without ribavirin who developed on-treatment elevation in AST/ALT, all of whom demonstrate a clinical scenario similar to that described for women on OCPs, and all of whom achieved SVR with treatment.

## 2. Case Presentation

### 2.1. Case  1

**Figure 1:**
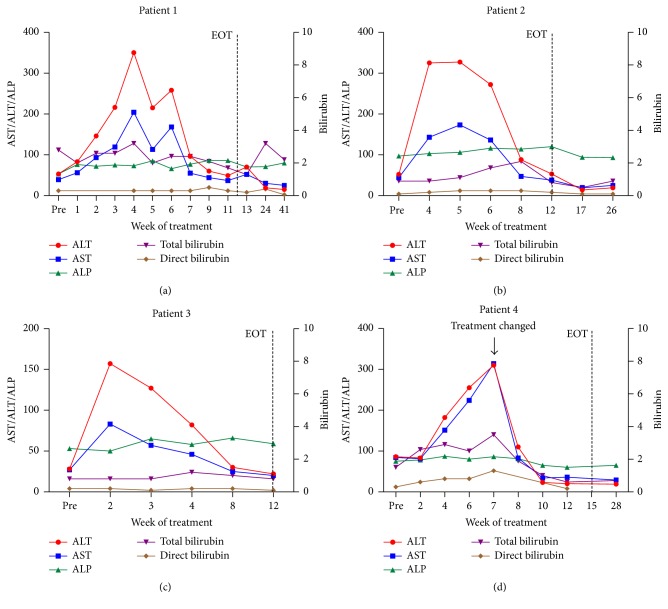
Liver enzyme progression for individual patients. EOT: end of treatment; ALP: alkaline phosphatase.

A 62-year-old presented with treatment-naïve, HCV genotype 1b infection, and well-controlled HIV coinfection on a regimen of emtricitabine, tenofovir, atazanavir, and ritonavir with a CD4 count of 580 cells/mm^3^ and an HIV viral load < 40 copies/mL. Liver biopsy in 2004 revealed stage 0 disease, liver staging by Fibrosure in 2012 suggested F3-F4 disease (measured while taking atazanavir), and a 1.9 cm liver biopsy in 2013 again revealed stage 0 disease. Additional noninvasive staging revealed a FIB4 score of 2.3 and an APRI score of 0.89. Ultrasonography revealed normal liver size and echogenicity, with no evidence of portal hypertension or splenomegaly. Baseline labs included HCV viral load 61,251 IU/mL, platelets 158,000/mm^3^, ALT 83 IU/mL, AST 56 IU/mL, albumin 4.0 g/dL, and total bilirubin 2.0 IU/mL (bilirubin elevation likely due to atazanavir). He had no other significant past medical history or medication use, other than receiving azithromycin for chlamydia urethritis 3 days prior to initiating PrOD therapy for 12 weeks without ribavirin. Safety labs obtained at week 1 of treatment revealed an elevation in AST/ALT over pretreatment values (Figure 1(a)). During continued treatment, he remained asymptomatic and had frequent laboratory checks, including direct bilirubin to monitor for drug-induced liver injury, as baseline indirect bilirubin was elevated due to atazanavir. By week 7 hepatic transaminases had declined markedly from their peak (Figure 1(a)). No medication modifications were made during PrOD treatment, and he achieved SVR 12 weeks after treatment with liver enzymes in the normal range.

### 2.2. Case  2

A 61-year-old presented with treatment-naïve, HCV genotype 1a monoinfection with liver biopsy 3 years prior to treatment revealing stage 1-2 fibrosis and FibroScan 2 months prior to treatment showing a stiffness score of 9.5 kPa, suggesting F2-F3 disease. Additional noninvasive staging revealed a FIB4 score of 2.3 and an APRI score of 0.68. Baseline labs included HCV viral load 11.6 million IU/mL, platelets 162,000/mm^3^, ALT 52 U/L, AST 44 U/L, albumin 4.4 g/dL, and total bilirubin 0.9 mg/dL. Past medical history included spinal fusion surgery, hypertension (not on medications), and hyperlipidemia (on pravastatin 40 mg daily, a dose that did not require modification) [1]. PrOD was initiated for 12 weeks with weight-based ribavirin. At week 4 of treatment, an elevation in AST/ALT over baseline values was observed (Figure 1(b)). He reported mild side effects from therapy including fatigue, headache, insomnia, and itching that all resolved by week 4 of treatment. PrOD and ribavirin therapy were continued, he remained asymptomatic, and 12 weeks after treatment AST/ALT were in the normal range and HCV RNA was undetectable, indicating SVR.

### 2.3. Case  3

A 63-year-old presented with treatment-naïve, genotype 1a HCV monoinfection, with liver biopsy 4 years prior to treatment revealing stage 2-3 disease. Prior to treatment, noninvasive staging revealed a FIB4 score of 1.6 and an APRI score of 0.35. Baseline labs included HCV viral load 1.1 million IU/mL, platelets 194,000/mm^3^, ALT 28 U/L, AST 27 U/L, albumin 4.1 g/dL, and total bilirubin 1.5. Past medical history included hypertension, for which he took amlodipine and hydrochlorothiazide. PrOD was initiated for 12 weeks with weight-based ribavirin. At week 2 of treatment, labs revealed an elevation in AST/ALT over baseline values (Figure 1(c)). He was asymptomatic and treatment was continued without dose modification. By week 3, hepatic transaminases were declining and normalized by week 8. Hemoglobin and hematocrit remained stable throughout the treatment course and the patient achieved SVR.

### 2.4. Case  4

An 82-year-old presented with treatment-naïve, genotype 1a HCV monoinfection with FibroScan 4 months prior to treatment revealing a stiffness score of 14 kPa, suggesting cirrhosis. Noninvasive staging scores were FIB4 4.6 and APRI 1.3. Liver ultrasound revealed changes consistent with cirrhosis and no concerning masses. Pretreatment labs included HCV viral load 46,382 IU/mL, platelets 160,000/mm^3^, ALT 86 U/L, AST 84 U/L, albumin 3.6 g/dL, and total bilirubin 1.5 U/L. Past medical history included hypertension (on hydrochlorothiazide), chronic idiopathic peripheral neuropathy (on gabapentin), and cecal adenocarcinoma treated surgically. Patient evaluation at treatment initiation did not reveal any signs of decompensated cirrhosis. Treatment with PrOD with weight-based ribavirin was initiated. At week 2, safety labs revealed unchanged AST/ALT values and a slight increase in total bilirubin (Figure 1(d)), as well as a decline in hemoglobin and hematocrit from 15.3/45.2 g/L to 13.5/38.3 g/L, respectively. He was asymptomatic and treatment was continued without dose modification. By week 4 of treatment, AST/ALT had increased while total bilirubin remained stable (Figure 1(d)) and he reported mild foot swelling, not present prior to treatment initiation. Physical exam confirmed bilateral 1+ pitting edema on the dorsum of his feet. At week 5 of treatment, hemoglobin and hematocrit had declined to 11.7/35.4, respectively, and bilateral pedal edema had increased to 2+. Ribavirin dose was reduced from 1200 mg/day to 600 mg/day. By week 7 of treatment, AST/ALT had increased further (Figure 1(d)), hemoglobin and hematocrit remained stable, and creatinine had increased from 1.34 at baseline to 2.15 mg/dL. He remained asymptomatic and had stable foot swelling that was relieved by use of compression stockings. PrOD and ribavirin therapy were stopped and the patient continued HCV therapy with ledipasvir-sofosbuvir. At week 10 of total HCV therapy (week 3 of ledipasvir-sofosbuvir) serum creatinine and AST/ALT had returned to baseline and remained normal thereafter (Figure 1(d)). He completed a total of 8 weeks of ledipasvir-sofosbuvir following the 7 weeks of PrOD and ribavirin. Foot swelling persisted throughout treatment but had resolved by 28 weeks after initiation of treatment when SVR was achieved.

## 3. Discussion

Herein, we present a detailed clinical description of AST/ALT elevations that can occur during PrOD therapy, with or without ribavirin, for chronic HCV genotype-1 infection. Although caution is recommended with concomitant use of estradiol-containing oral contraceptive medications [1], all 4 subjects in our series were African-American males with advancing age and differing degrees of hepatic fibrosis, including 1 patient with cirrhosis. All completed HCV treatment, with therapy modification in 1 case, and all achieved SVR. No medications or comorbidities were shared amongst these patients in terms of an alternate etiology for the observed pattern of hepatic transaminases.

Aside from a higher risk of AST/ALT elevations reported in women taking concomitant estrogen therapy during PrOD therapy, there are no reports of other specific patient factors related to an increased incidence of enzyme alterations that may help clinicians target patients for close monitoring or anticipate liver enzyme elevations. This report indicates and suggests that these abnormalities can occur in African-American men with advanced age and highlights the importance of close lab monitoring during treatment in cirrhotic patients in light of new FDA warnings for risk of liver toxicity with PrOD use. To determine whether age, race, and gender are significant risk factors for ALT elevation during PrOD therapy would require observation in a larger cohort of treated patients. Of note, none of our patients had a liver enzyme pattern suggestive of drug-induced liver injury. For case 4, therapy was switched during the peak of AST/ALT elevation and within the reported typical window of lab abnormality resolution, and thus it is challenging to know whether hepatic transaminases would have resolved without a switch in therapy.

In summary, these cases suggest that all PrOD patients should have monitoring as early as week 2 of treatment to monitor for abnormalities, with a particular emphasis on early monitoring in cirrhotic patients. While the patients with advanced liver disease who experienced liver injury associated with PrOD suggested that elevated transaminases were not as prominent a feature of their presentation as that reported in the package insert [6], close monitoring in the setting of cirrhosis remains a recommendation. For HIV/HCV coinfected patients on antiretroviral therapy containing atazanavir, direct bilirubin should be utilized to monitor for drug-induced liver injury given elevations in indirect bilirubin as a result of atazanavir use. Importantly, these cases also highlight that AST/ALT elevations for those without cirrhosis did not have an apparent clinical consequence and all patients progressed to achieve SVR.
